# Going viral: lessons from an accelerated PPE teaching programme during the COVID-19 pandemic

**DOI:** 10.15694/mep.2020.000176.1

**Published:** 2020-08-26

**Authors:** Benjamin Bennett, Silas Fuller, Patrick Howlett, Alanna Hare

**Affiliations:** 1 Royal Brompton Hospital

**Keywords:** iterative learning, interprofessional education, multiprofessional simulation-based education, COVID-19, coronavirus, PPE

## Abstract

This article was migrated. The article was marked as recommended.

**Introduction:** at the beginning of the COVID-19 pandemic in the United Kingdom, there was an urgent need to establish a teaching programme to rapidly upskill hospital staff in the use of Personal Protective Equipment (PPE).

**Aims:** to train all members of clinical and non-clinical staff operating within the respiratory department of the Royal Brompton Hospital over a period of one week, using a series of multi-professional simulation-based sessions and to then share the learning points gained in order to assist others facing the same issue.

**Results:** using an iterative style and a mastery learning model, the designated teaching faculty rapidly implemented a situated teaching programme, managing to train all staff members within the given timeframe. Given the time-critical nature of the programme, sessions required considerable flexibility to change to fit a learner-driven agenda, drawing benefits from an andragogical approach and an emphasis on interprofessional learning.

## Introduction

At the start of the COVID-19 pandemic, all staff working within the respiratory department of the Royal Brompton Hospital (RBH), London, required training in the use of Personal Protective Equipment (PPE). To be effective, this training needed to encompass all staff members, including non-clinical staff; take place rapidly before the peak of the pandemic and consider the risk of transmission between staff during teaching. This programme was designed to take place over a one-week period to ensure that staff were competent in the use of PPE before a significant risk of exposure developed.

## Methods

Information was collated from local and national guidelines and expert local opinion sought from the Infection Prevention and Control (IPC) team to train a large teaching faculty in applying the donning, doffing and testing guidelines outside a specialist IPC environment. This allowed for rapid information dissemination and adequate cross-cover for sickness at a presumed rate of one third of faculty at any one time. This was based upon emerging data from the Royal College of Physicians suggesting that approximately 30% of London-based doctors had taken time off work since the arrival of COVID-19 in the UK ( Royal College of Physicians, 2020). Teaching faculty were freed from their usual duties to run continuous 30-minute sessions throughout the day. A multi-professional simulation-based approach was chosen, training all staff in mixed groups in a ward-based environment within a ‘clean’ area of the hospital. Attendees were capped to a total of six persons per room in each session to enable social distancing. In order to enhance the situated learning aspect of the training, a simulation dummy was used to increase fidelity, with simulated low infection risk and high infection risk (aerosol-generating) procedures interwoven with learning of the COVID-19 case-definition and viral testing scenarios for those staff in a clinical role. All techniques were first demonstrated by a faculty member and learners then practiced all relevant procedures themselves using a mastery learning model which offered opportunity for deliberate practice with faculty feedback to consolidate learning and achieve a consistent “mastery” standard, ensuring all learners accomplished all educational objectives ( McGachie *et al.,* 2009).

## Learning Points

The programme was designed to function in an iterative style, allowing for a dynamic approach in addressing issues raised during the sessions. Key to this were short daily debriefs between faculty and the IPC team, covering new questions or problems identified and any required changes to the teaching programme; as well as any issues identified which required changes to be made in the clinical environment.

The most prominent learning point in the initial implementation was that the session was too content-heavy. It immediately became clear in clinical practice that the first sessions had not been fully absorbed, so consequently, multiple sessions were introduced, first teaching basic principles of PPE usage in a low-fidelity setting and then increasing fidelity by adding tailored clinical scenarios into subsequent sessions. It was found that in such a time-pressured environment, a truly high-fidelity approach had to be sacrificed in the interest of a learner-derived agenda. While benefit was reported from the situation of the learning in a ward environment, it proved more important to the learners to all have the opportunity for deliberate practice to achieve “mastery” standard with faculty feedback than to focus on formal simulation training as initially planned.

One measure which was introduced during the programme and proved successful was the identification of volunteers for a ‘PPE champion’ role in each department,. The aim of this change was to move closer to the principles of adult learning ( Knowles, 1984). Once introduced, this innovation did help to flatten the classroom hierarchy, allowing the learners to transition effectively to a more andragogical peer-led learning process. This approach also proved beneficial in addressing concern over the rapidly changing guidance surrounding PPE. It was emphasised to learners that the basic skills they had learned would remain unchanged, but having been signposted to appropriate resources, they would take the responsibility in remaining up-to-date with new guidance and applying their prior knowledge accordingly.

More immediate successes were also seen, particularly the benefit of interprofessional learning, with enthusiastic verbal feedback from allied health professionals emphasising the sense of togetherness fostered by the shared uncertainty and learning with, from and about one another ( Young *et al.,* 2007). The range of professionals within each group resulted in a series of varied questions, each from the perspective of their respective professions. This proved extremely useful for both interprofessional education and accelerated the adaptive cycle by identifying points which had hitherto not been considered.

In summary, we hope that others may benefit from the knowledge gained here in the delivery of “just-in-time” training through simple, learner-focused sessions, at a time when speed was critical.

## Take Home Messages

• Consider the risk of COVID-19 transmission when designing a programme to minimise the risk posed to staff by attending.
• An iterative style ensures that time-critical sessions can begin immediately: it is important not to wait until a session is perfect before commencing training.
• A system of regular feedback is vital to ensure that a teaching programme can respond to a rapidly evolving crisis and remain relevant to the needs of the learners.
• Allowing learners to drive their own agenda with a problem-orientated approach will often result in improved engagement in comparison to a purely content-orientated approach, improving the desired outcome.

## Notes On Contributors

Dr Benjamin Bennett is an IMT1 trainee at the Royal Brompton Hospital who began his journey into medical education following completion of a PGCert qualification in 2019. https://orcid.org/0000-0002-0913-8255

Dr Silas Fuller is an IMT1 trainee at the Royal Brompton Hospital who in turn is planning to enter a PGCert course in 2020. https://orcid.org/0000-0002-0783-7757

Dr Patrick Howlett is a respiratory registrar in the North West Thames deanery with an interest in infection and epidemiology. https://orcid.org/0000-0003-2381-7067

Dr Alanna Hare is a consultant respiratory physician at the Royal Brompton Hospital, subspecialising in Sleep and Ventilation. In addition to her clinical duties, she has held a number of educational roles and is currently the Royal College of Physicians tutor at her trust.

## Declarations

The author has declared that there are no conflicts of interest.

## Ethics Statement

No ethics approval was required as this is not an original research project.

## External Funding

This article has not had any External Funding
