# Supplementary material for: Going viral: lessons from an accelerated PPE teaching programme during the COVID-19 pandemic
Source: MedEdPublish (2016). 2020 Aug 26;9:176. [Version 1] doi: 10.15694/mep.2020.000176.1 (PMC10702669; doi:10.15694/mep.2020.000176.1)
Supplement: Peer review history

# Reviewer report: Reviewer response for version 1

**DOI:** 10.21956/mep.20124.r31050

**Published:** 2020-11-14

**Authors:** Ken Masters

**Affiliations:** 1 Sultan Qaboos University

This review has been migrated. The reviewer awarded 3 stars out of 5 An interesting short paper on lessons from an accelerated PPE teaching programme during the COVID-19 pandemic.While the paper does have value, in the absence of an evaluation, greater value would be derived if the authors could supply more detail about the course, and how it changed. There is an overall broad description in the paper, but a view of the instructions/guidelines - at the beginning of the training and then once changes had been made – would prove to be more beneficial to the reader.These could be supplied as appendices with appropriate commentary.

Reviewer Expertise:

NA

No decision status is available

# Reviewer report: Reviewer response for version 1

**DOI:** 10.21956/mep.20124.r31052

**Published:** 2020-08-27

**Authors:** Trevor Gibbs

**Affiliations:** 1 AMEE

This review has been migrated. The reviewer awarded 4 stars out of 5 An interesting, short but practical paper that I feel the content of which will be part of our new normal in undergraduate / postgraduate education and faculty development. Written with the idea of making sure that the training was based upon educational theory, it was refreshing to hear of both the difficulties and successes, although I feel that this section might have been a little more expansive and explorative. I would recommend this paper to all involved with health professional education.

Reviewer Expertise:

NA

No decision status is available

# Reviewer report: Reviewer response for version 1

**DOI:** 10.21956/mep.20124.r31051

**Published:** 2020-08-26

**Authors:** Carol Quinn

**Affiliations:** 1 University of Glasgow

This review has been migrated. The reviewer awarded 4 stars out of 5 A useful article with practical implications for various medical training programmes. The 'just in time' model described captures the essence of identifying effective learning styles and the importance of flexibility and adaptiveness in delivering training to medical staff within the context of a high stress working environment.

Reviewer Expertise:

NA

No decision status is available
